# Silibinin reduces *in vitro* methane production by regulating the rumen microbiome and metabolites

**DOI:** 10.3389/fmicb.2023.1225643

**Published:** 2023-08-23

**Authors:** Rui Liu, Yueyu Shen, Haokai Ma, Yang Li, Modinat Tolani Lambo, Baisheng Dai, Weizheng Shen, Yongli Qu, Yonggen Zhang

**Affiliations:** ^1^College of Animal Science and Technology, Northeast Agricultural University, Harbin, China; ^2^Beijing Sunlon Livestock Development Company Limited, Beijing, China; ^3^College of Electrical Engineering and Information, Northeast Agricultural University, Harbin, China; ^4^College of Animal Science and Technology, Heilongjiang Bayi Agricultural University, Daqing, China; ^5^Key Laboratory of Low-carbon Green Agriculture in Northeastern China of Ministry of Agriculture and Rural Affairs, Daqing, China

**Keywords:** silibinin, methane, rumen fermentation, microbiome, metabolite

## Abstract

This study used Silibinin as an additive to conduct fermentation experiments, wherein its effects on rumen gas production, fermentation, metabolites, and microbiome were analyzed *in vitro*. The silibinin inclusion level were 0 g/L (control group), 0.075 g/L, 0.15 g/L, 0.30 g/L, and 0.60 g/L (experimental group). Fermentation parameters, total gas production, carbon dioxide (CO_2_), methane (CH_4_), hydrogen (H_2_), and their percentages were determined. Further analysis of the rumen microbiome’s relative abundance and α/β diversity was performed on the Illumina NovaSeq sequencing platform. Qualitative and quantitative metabolomics analyses were performed to analyze the differential metabolites and metabolic pathways based on non-targeted metabolomics. The result indicated that with an increasing dose of silibinin, there was a linear reduction in total gas production, CO_2_, CH_4_, H_2_ and their respective percentages, and the acetic acid to propionic acid ratio. Concurrent with a linear increase in pH, when silibinin was added at 0.15 g/L and above, the total volatile fatty acid concentration decreased, the acetic acid molar ratio decreased, the propionic acid molar ratio increased, and dry matter digestibility decreased. At the same time, the relative abundance of *Prevotella*, *Isotricha*, *Ophryoscolex*, *unclassified_Rotifera*, *Methanosphaera*, *Orpinomyces*, and *Neocallimastix* in the rumen decreased after adding 0.60 g/L of silibinin. Simultaneously, the relative abundance of *Succiniclasticum*, *NK4A214_group*, *Candidatus_Saccharimonas*, and *unclassified_Lachnospiraceae* increased, altering the rumen species composition, community, and structure. Furthermore, it upregulated the ruminal metabolites, such as 2-Phenylacetamide, Phlorizin, Dalspinin, N6-(1,2-Dicarboxyethyl)-AMP, 5,6,7,8-Tetrahydromethanopterin, Flavin mononucleotide adenine dinucleotide reduced form (FMNH), Pyridoxine 5′-phosphate, Silibinin, and Beta-D-Fructose 6-phosphate, affecting phenylalanine metabolism, flavonoid biosynthesis, and folate biosynthesis pathways. In summary, adding silibinin can alter the rumen fermentation parameters and mitigate enteric methane production by regulating rumen microbiota and metabolites, which is important for developing novel rumen methane inhibitors.

## 1. Introduction

As the global population continues to rise, more than a third of the protein demand for humans, such as meat and milk, depends on animal agriculture, thus resulting in livestock production intensification and consequently causing the agricultural sector to be a major source of greenhouse gas (GHG) generation (Sakadevan and Nguyen, 2017). The main greenhouse gas emitted from the animal sector is carbon dioxide (CO_2_), methane (CH_4_)_,_ and nitrous oxide (N_2_O). Controlling GHG emissions is key to stabilizing the global climate system because its excess in the atmosphere leads to frequent natural disasters, increases the spread of pathogenic diseases, and poses a risk to the health and survival of humans and animals alike. The contribution of methane to global warming is more prominent than N_2_O because it constitutes about half of the entire greenhouse gas in the atmosphere (Stocker et al., 2013; Myhre et al., 2014; Haque, 2018), with most of the methane emanating from ruminants. Therefore, reducing CH_4_ emissions from enteric fermentation in ruminants is an effective way to mitigate greenhouse gas emissions in the livestock industry (Vaghar Seyedin et al., 2022).

CH_4_ produced by ruminants is mainly from the enteric fermentation process initiated by rumen microorganisms through the CO_2_ reduction pathway under the action of methanogenic bacteria while reducing the partial buildup of rumen H_2_, maintaining the stability of rumen pH and ensuring normal rumen fermentation and the methane produced is released into the atmosphere through feces or burp. Therefore, it is crucial to mitigate CH_4_ by regulating the rumen microbiota. Research has found that adding ionic carriers such as monensin to the diet could alter the rumen microbiome, inhibit the production of available substrates by methanogenic bacteria, and reduce CH_4_ production (Marques and Cooke, 2021). However, with longer feeding times, ion carriers could develop resistance in some bacteria (Vendramini et al., 2016) and have therefore been banned in many countries. The addition of methanogenic inhibitors such as nitro derivatives, on the other hand, though could reduce CH_4_ production *via* rumen microbiome alteration, but there are very few reports on their safety assessment (Teng and Kim, 2021).

Plant extracts are gaining popularity due to their safe and non-toxic benefits. Literature has reported that plant extracts have colony-modulating effects (Jubair et al., 2021; Vaou et al., 2021) and that flavonoids extracted from plants not only reduce the number of methanogenic bacteria in the rumen but also reduce the number of protozoa attached to the methanogens to reduce CH_4_ production (Ku-Vera et al., 2020). Seradj et al. (2014) observed that adding flavonoid extract inhibited *hydrogenotrophic methanogenic archaea* and *Methanosarcina* in the rumen. Likewise, the flavonoid-rich extract obtained from Brazilian spinach (*Alternanthera sissoo*; BS) was found to decrease CH_4_ production and protozoa counts while elevating total volatile fatty acid and propionic acid levels during *in vitro* fermentation (Sommai et al., 2021).

Silibinin (C_25_H_22_O_10_), an active ingredient isolated from the fruit of *Silybum marianum* (chrysanthemum plant), is single in composition and chemistry as a dihydro flavonol compound compared to some other flavonoids extracted from plants. According to recent scientific investigations, gram-positive bacteria are more susceptible to the inhibitory effects of silibinin as compared to their gram-negative counterparts (Janssen and Kirs, 2008; Miller, 2015; Mahmoudi-Rad et al., 2022). Therefore, the potential of silibinin to effectively regulate the activity and population of gram-positive bacteria associated with methane synthesis is critical for methane regulation. Additionally, the mechanism by which silibinin regulates gram-positive bacteria is mainly by inhibiting protein and RNA synthesis (Lee et al., 2003). Some reports have shown that silibinin has a modulating effect on the activity of *Staphylococcus aureus* (Cai et al., 2018), *Bacillus cereus* (Mahmoudi-Rad et al., 2022), and *Prevotella intermedia* (Lee et al., 2012). Also, its anti-inflammatory effects are exerted by regulating the microbial flora (Rakelly de Oliveira et al., 2015).

Even though flavonoids have been shown to have regulatory effects on rumen microflora and amino acids metabolic pathways such as tyrosine metabolism and proline metabolism (Yu et al., 2023), there are no studies on the use of silibinin as an additive in dairy cows. Based on the above, this study hypothesized that silibinin could affect rumen fermentation by regulating rumen microbiota and metabolites, thereby reducing methane production. Therefore, using *in vitro* fermentation method, we assessed rumen fermentation characteristics and gas production, analyzed the relative abundance and diversity of the rumen microbiome, and revealed the metabolites and metabolic pathways involved. This experiment provides a sufficient theoretical basis and data support for the application of silibinin in regulating ruminal methanogenesis in ruminants.

## 2. Materials and methods

### 2.1. Animals, diet, and experimental design

The animal use protocol was approved following the Animal Care and Use Committee of Northeast Agricultural University (protocol number: NEAUEC20230268). Three lactating Chinese Holstein cows (day in milk = 120 ± 11, milk yield = 27.6 ± 3.3 kg) were housed individually in a tethered barn stall, and water was made available freely. All cows were fed the same amount of total mixed ration (TMR) and milked twice daily at 0630 and 1830. The ingredients and chemical composition of the experimental diet are indicated in Table 1.

**Table 1:** Composition and nutrient levels of the basal diet (DM basis).

Items	Contents %
Alfalfa hay	15.59
Corn silage	30.28
Soybean meal	11.14
Flaked corn	19.15
Dried corn husk	5.35
Dried distillers grains with solubles (DDGS)^a^	5.35
Sugar beet pellets	12.03
Premix^b^	1.11
Nutrient composition	
CP^c^, %	16.67
NE_L_^d^, MCal/kg	1.72
RDP^e^, %CP	60.35
ADF^f^, %	21.11
NDF^g^,%	32.75
peNDF^h^, %	22.95
Starch,%	27.12
Crude fat^i^, %	4.62
Ash, %	7.14
Ca, %	0.61
P, %	0.59

a DDGS contained (on a DM basis): 88.7% DM, 27.6% CP, 28.4% NDF, 23.5% ADF, 3.11% ether extract, 0.76% Lys, 0.67% Met.

b Premix = The premix contained (on a DM basis): 99.17% ash, 14.25% Ca, 5.40% P, 4.93% Mg, 0.05% K, 10.64% Na, 2.95% Cl, 0.37% S, 12 mg/kg Co, 500 mg/kg Cu, 4,858 mg/kg Fe, 25 mg/kg I, 800 mg/kg Mn, 10 mg/kg Se, 1800 mg/kg Zn, 180,000 IU/kg vitamin A, 55000 IU/kg vitamin D and 1,500 IU/kg vitamin E.

c CP = Crude protein.

d NE_L_ = Net Energy for Lactation (Wei et al., 2018).

e RDP = Rumen degradable protein.

f ADF = Neutral detergent fiber.

g NDF = Neutral detergent fiber.

h peNDF = Physically effective neutral detergent fiber (Yang and Beauchemin, 2006).

i Crude fat determination (ZeidAli-Nejad et al., 2018).

The anaerobic culture techniques and experimental procedures were similar, as described by Xin et al. (2021). Rumen fluid, sampled from the three fistulated cows before morning feeding, was flushed with O_2_-free CO_2_ in a vacuum flask, sieved through a four-layered cheesecloth before mixing with pre-warmed (39°C) buffer, and prepared according to (Soliva and Hess, 2007), at a ratio of 1:2 (buffer: ruminal fluid, v:v) under a continual CO_2_ flow to maintain an anaerobic environment. After mixing, 150 mL was transferred to a 200 mL glass bottle containing 2 g fermentation TMR substrates. The silibinin, purchased from Daxing’anling Livorcom Biotechnology Co., Ltd. China (purity of silibinin: 98%), with different qualities, was added to the rumen fluid to make 0.00, 0.075, 0.15, 0.30, and 0.60 g/L final concentrations; the blank control glass bottle only contained mixed abomasum fluid. Silicone stoppers with gas-tight collection bags were used to seal the sample bottles (ten replicates per treatment) before incubating them in a shaking water bath at 39°C and 40–50 rpm for 24 h. These incubations were performed in three separate runs over three days.

### 2.2. Total, hydrogen, carbon dioxide and methane gas production, and rumen fermentation analysis

During fermentation, the total gas produced was collected using gas collection bags. Each gas collection bag was connected to the top of the fermentation bottle *via* a rubber tube, and after ensuring that the entire device was airtight, incubation was carried out. At the end of the incubation period, the volume of the gas samples was measured using a syringe. Subsequently, a 0.5-mL subsample of the gas was analyzed for its hydrogen, carbon dioxide, and methane content using a Gas chromatograph (GC-8A; Shimadzu Co. Ltd., Tokyo, Japan) with a molecular sieve 13×, 45- to 60-mesh column (2.0 mm × 3.2 mm × 2.0 mm, stainless steel), and thermal conductivity detector. The temperature was set to 60°C, injector and thermal conductivity detector temperatures were set at 120°C, and flame-ionization detector temperature was set at 200°C. The carrier gas (N_2_) flow rate was 50 mL/min, in line with the procedure outlined by Kim et al. (2014). The pH level of the culture fluid was then measured with a pH meter (Sartorius basic pH meter, Göttingen, Germany). The rumen culture liquor was filtered through a 4-layered cheesecloth and stored at −20°C with 2 mL metaphosphoric acid (25%, wt/vol) per 10 mL before determining the VFA and ammonia-N levels. The VFA concentration was determined using gas chromatography (GC-8A; Shimadzu Corp., Kyoto, Japan) as described by Hu et al. (2005). Meanwhile, ammonia-N levels were determined using the phenol-hypochlorite method (Broderick and Kang, 1980).

### 2.3. Dry matter digestibility analysis

2 g of TMR sample was weighed into the fiber bag as a fermentation substrate. Ruminal fluid and buffer were mixed in a ratio of 1:2 as described above and added to the glass flask before placing it in the fiber bag. Air bubbles in the glass flasks were removed before sealing and then incubated in a constant temperature water bath at 39°C with shaking. After continuous fermentation for 24 h, the fiber bag was removed, rinsed with distilled water three times, dried for 48 h at 55°C, and then cooled in a desiccator (Kim et al., 2019). The sample was then weighed and used to determine the fermentation substrate’s *in vitro* dry matter digestibility (IVDMD), according to Wang et al. (2017).

IVDMD (%) = (DM content before fermentation – DM content after fermentation)/ DM content before fermentation × 100%.

### 2.4. DNA extraction, sequencing, and diversity analysis

At the end of the incubation, 20 mL of culture liquor with a maximum dose of 0.60 g/L of silibinin was split into two equal portions before storing at −80°C for 16S amplicon sequencing and untargeted metabolomics analysis.

The procedure involved the extraction of total DNA from the culture liquor using QIAamp Fast DNA stool Mini Kit (Qiagen, Cat# 51604) followed by the amplification of PCR conducted with barcoded specific bacteria primers that targeted the variable region 3–4 (V3-V4) of the 16S rRNA gene: forward primer 338F: 5′- ACTCCTACGGGAGGCAGCA -3′ and reverse primer 806R: 5′- GGACTACHVGGGTWTCTAAT -3′ (Wang et al., 2023). The V3-V4 region of the 16S rRNA gene for archaeal analysis was amplified using primers F341 (5′- GYGCASCAGKCGMGAAW -3′) and R806 (5′- GGACTACVSGGGTATCTAAT -3′). The protozoa analysis involved amplifying the V3-V4 region of the 18S rRNA gene using TAReuk454FWD1 F (5′- CCAGCASCYGCGGTAATTCC -3′) and TAReukREV3 R (5′- ACTTTCGTTCTTGATYRA -3′) primers. For fungi analysis, the ITS region of the 16S rRNA gene for fungi was amplified using primers F (CTTGGTCATTTAGAGGAAGTAA) and R (GCTGCGTTCTTCATCGATGC). According to standard protocols, sequencing libraries and paired-end sequencing was constructed on an Illumina NovaSeq6000 platform at Biomarker Technologies Co, Ltd. (Beijing, China). Following Yang et al. (2020), paired-end reads were merged using FLASH v1.2.7, and tags with over six mismatches were ignored. Trimmomatic (Sheng et al., 2019) was used to identify merged tags having an average quality score of <20 in a 50 bp sliding window, and tags shorter than 350 bps were eliminated. Using USEARCH, potential chimeras were eliminated, and the denoised sequences were grouped into operational taxonomic units (OTUs) with 97% similarity (version 10.0). All OTUs were given a taxonomy *via* a QIIME search against the Silva databases (Release 128).

The microbiome was compared as beta diversity using partial least squares discriminant analysis (PLS-DA). Taxa abundance at the genus level was statistically compared between the control and treatment groups for exposure to silibinin and the ruminal microbiome abundance.

### 2.5. Non-targeted metabolomics analysis

For quality control purposes, 10 μL per sample of the prepared rumen fluid was pooled. Metabolomics analysis was conducted using a Waters Acquity I-Class PLUS ultra-high performance liquid tandem and Waters Xevo G2-XS QTOF high-resolution mass spectrometer with a Waters Acquity UPLC HSS T3 (1.8um, 2.1 × 100 mm) column as outlined by Zhang et al. (2021). The injection volume for both positive and negative modes was set at 1 μL, with mobile phase A consisting of 0.1% formate aqueous solution and mobile phase B containing 0.1% formate acetonitrile.

After software acquisition control using MassLynx V4.2 (Waters), the primary and secondary mass spectrometry data were extracted using a Waters Xevo G2-XS QTOF high-resolution mass spectrometer in MSe mode. Dual-channel data acquisition was performed, alternating between a low collision energy of 2 V and a high collision energy of 10–40 V per cycle with a scanning frequency of 0.2 s for a mass spectrum, as described by Feng et al. (2022). The ESI ion source utilized a capillary voltage of 2,000 V (positive ion mode) or −1,500 V (negative ion mode), a 30 V cone voltage, a 150°C ion source temperature, 500°C desolvent gas temperature, 50 L/ h backflush gas flow rate, and 800 L/h desolventizing gas flow rate. Data processing operations such as peak extraction, peak alignment, and identification were performed using the online METLIN database and Biomark’s custom identification library with theoretical fragment identification and mass deviation calculations under 100 ppm, all utilizing Progenesis QI software.

The positive and negative data were combined, and the dependability of the model was assessed through 200 permutations after normalizing the original peak area data using principal component analysis (PCA) and orthogonal projections to latent structures-discriminant analysis (OPLS-DA) analysis. Following the grouping information, the difference multiples were calculated and compared. Compounds identified were classified, and pathway information was searched against KEGG databases. Additionally, a T-test was utilized to determine each compound’s differential significance *p* value, with multiple cross-validations used to calculate the OPLS-DA model’s VIP value. The differential metabolites were then filtered at FC > 1 or < −1, *p* value <0.05, and VIP > 1 using a technique that integrates the difference multiple, *p* value, and VIP value of the OPLS-DA model. A hypergeometric distribution test was conducted to determine different metabolites for KEGG pathway enrichment significance. Volcano plots were generated using ggplot2 in R language to filter the metabolites of interest based on their log2 (Fold change) and - log10 (*p*-value).

### 2.6. Statistical analysis

All data from the experiment were analyzed using SAS software (Version 9.4; SAS Institute Inc., Cary, NC). Histograms and formal statistical tests included in the UNIVARIATE procedure of SAS were used to test for normal distribution and homogeneity of variance of gas production and concentration and rumen fermentation parameters. Data were then analyzed using a one-way ANOVA, and orthogonal polynomial contrasts were utilized to analyze the linear, quadratic, and cubic effects of silibinin under different concentrations. Relative abundances (%) of rumen microbiome *in vitro* after 24 h exposure to silibinin were analyzed using the non-parametric Kruskal-Wallis Test, and results were presented as mean with standard deviation. Significantly different means were declared when *p* ≤ 0.05, while trends were defined at 0.05 < *p* ≤ 0.10.

## 3. Results

### 3.1. Effect of silibinin on *in vitro* gas production

To assess the impact of silibinin addition on rumen fermentation, we employed an *in vitro* gas production technique to measure the corresponding fermentation products. After 24 h of *in vitro* fermentation, total gas production and CH_4_, CO_2_, and H_2_ production and ratio showed linear decreases (*p* < 0.001) with the increase in the amount of silibinin additive. Total gas production and CH_4_, CO_2_, and H_2_ production and ratios decreased to the lowest in the high dose 0.60 g/L group (Table 2).

**Table 2:** Effect of adding different doses of silibinin on gas production after 24 h of *in vitro* fermentation.

Items	Dose^1^(g/L)					SEM^2^	*p*-value		
	0.00	0.075	0.15	0.30	0.60		linear	quadratic	cubic
Total gas production, mL	143.20^a^	137.85^b^	131.65^c^	123.64^d^	119.46^d^	1.48	<0.001	0.37	0.89
Methane, mL	36.22^a^	31.25^b^	27.99^c^	24.90^d^	24.33^d^	0.33	<0.001	0.010	0.31
Carbon dioxide, mL	93.92^a^	86.69^b^	88.80^b^	78.09^c^	72.70^d^	0.94	<0.001	0.070	<0.001
Hydrogen, mL	1.13^a^	1.13^a^	0.97^b^	0.95^b^	0.91^c^	0.010	<0.001	0.36	<0.001
Methane /Total gas production, %	25.29^a^	22.67^b^	21.26^c^	20.14^d^	20.37^d^	0.19	<0.001	<0.001	0.29
Carbon dioxide /Total gas production, %	65.59^b^	67.45^a^	62.89^c^	63.16^c^	60.86^d^	0.52	<0.001	0.14	<0.001
Hydrogen /Total gas production, %	0.79^a^	0.82^ab^	0.74^b^	0.77^b^	0.76^b^	0.020	0.049	0.77	<0.001

^1^The amount of silibinin added was 0.00 g/L, 0.075 g/L, 0.15 g/L, 0.30 g/L, 0.60 g/L, respectively.

^2^SEM = Standarder error of mean.

^*^In the same row, values with different small letter superscripts mean significant difference (*p* ≤ 0.05), while with the same or no letter superscripts mean no significant difference (*p* > 0.05). The same as below.

### 3.2. Effect of silibinin on fermentation characteristics

With the increasing amount of silibinin additive, rumen pH and the molar ratio of propionic acid increased linearly (*p* < 0.001), and total volatile fatty acid concentration, acetic to propionic acid ratio, and dry matter digestibility decreased linearly (*p* < 0.001). With the addition of 0.075 g/L silibinin, NH_3_-N, total volatile fatty acid concentration, the molar ratio of acetic acid, the molar ratio of butyric acid, and dry matter digestibility were not statistically significant differences from the untreated group (Table 3). The result showed that adding low doses of silibinin did not affect rumen fermentation while reducing CH_4_ production.

**Table 3:** Effect of adding different doses of silibinin on rumen fermentation parameters after 24 h of *in vitro* fermentation.

Items	Dose(g/L)					SEM^1^	*p*-value		
	0.00	0.075	0.15	0.30	0.60		Linear	Quadratic	Cubic
pH	6.52^c^	6.54^b^	6.55^a^	6.55^ab^	6.55^a^	0.0040	<0.001	0.0020	0.57
Ammonia-N, mg/dL	47.07^a^	46.20^ab^	46.57^a^	46.68^a^	45.11^b^	0.41	0.66	0.23	0.40
Total volatile fatty acids, mmol/L	80.61^a^	79.06^a^	70.44^b^	62.03^c^	63.09^c^	2.51	<0.001	0.18	0.52
Acetic acid, mol/100 mol	56.44	53.78	53.07	55.55	54.00	1.23	0.54	0.042	0.82
Propionic acid, mol/100 mol	22.40^b^	23.50^b^	23.16^b^	24.84^ab^	25.70^a^	0.53	0.0055	0.59	0.15
Isobutyric acid, mol/100 mol	1.30^b^	1.38^ab^	1.47^a^	1.38^ab^	1.39^a^	0.053	0.26	0.12	0.45
Butyric acid, mol/100 mol	15.77	17.03	17.85	14.23	14.81	1.44	0.56	0.097	0.54
Isovaleric acid, mol/100 mol	2.25	2.37	2.43	2.15	2.16	0.13	0.72	0.14	0.64
Valeric acid, mol/100 mol	1.84	1.93	2.03	1.84	1.93	0.096	0.81	0.15	0.52
Acetic acid/propionic acid	2.52^a^	2.29^b^	2.30^b^	2.24^b^	2.10^c^	0.027	<0.001	<0.001	0.016
Dry matter digestibility, %	58.78^a^	59.19^a^	56.33^b^	56.06^b^	56.26^b^	0.35	<0.001	0.33	<0.001

^1^SEM = Standarder error of mean. In the same row, values with different small letter superscripts mean significant difference (*p* ≤ 0.05), while with the same or no letter superscripts mean no significant difference (*p* > 0.05).

### 3.3. Effect of silibinin on the rumen microbiome

**Figure 1:**
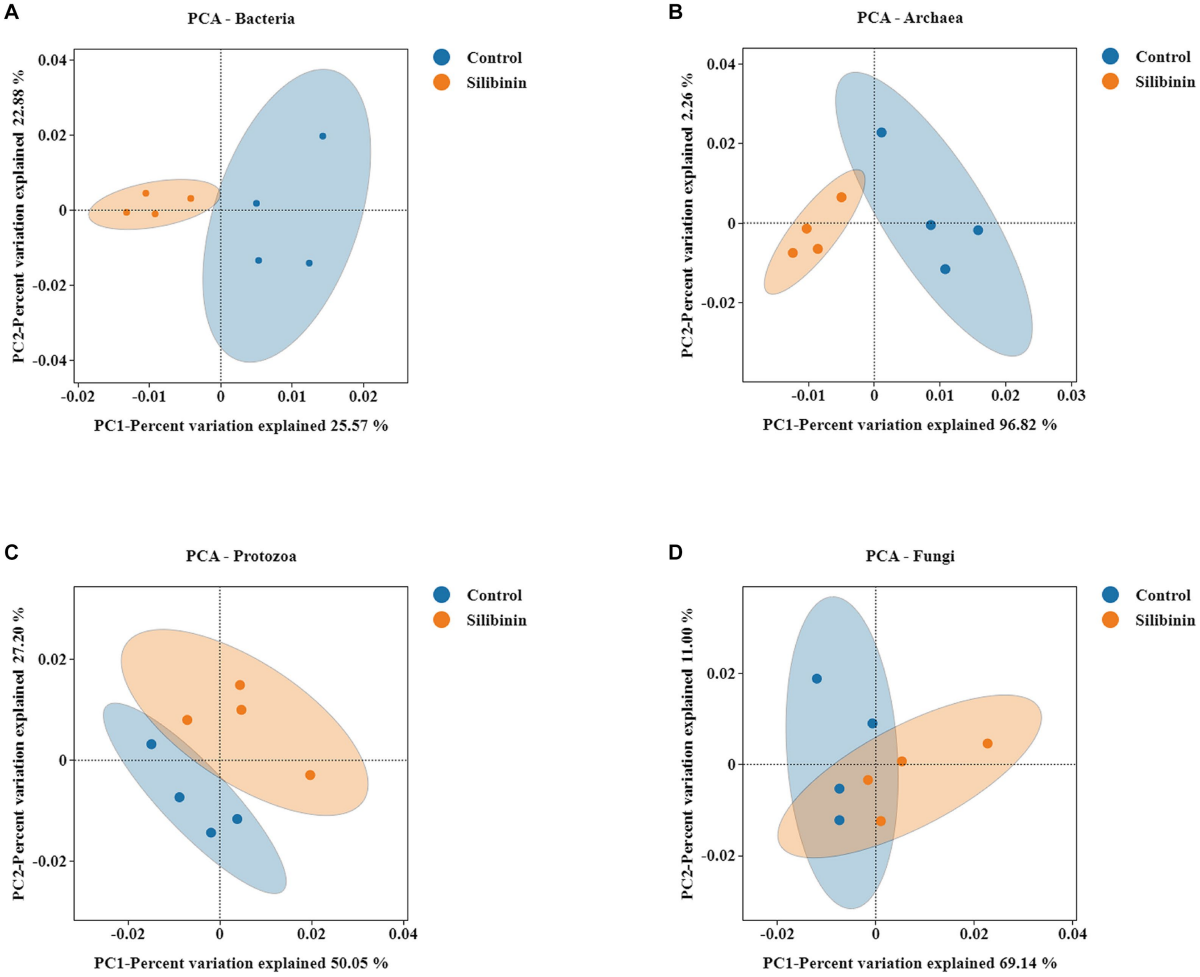
PCA analysis of rumen microbiome in the 0 g/L and 0.60 g/L silibinin addition group **(A)** Bacteria PCA analysis. **(B)** Archaea PCA analysis. **(C)** Protozoa PCA analysis. **(D)** Fungi PCA analysis.Each point in the figure represents a sample; different colors represent different groups; the oval circle indicates that it is a 95% confidence ellipse (that is, if there are 100 samples in the sample group, 95 will fall into it). The abscissa represents the first principal component, and the percentage represents the contribution value of the first principal component to the sample difference. The ordinate represents the second principal component, and the percentage represents the contribution value of the second principal component to the sample difference.

**Figure 2:**
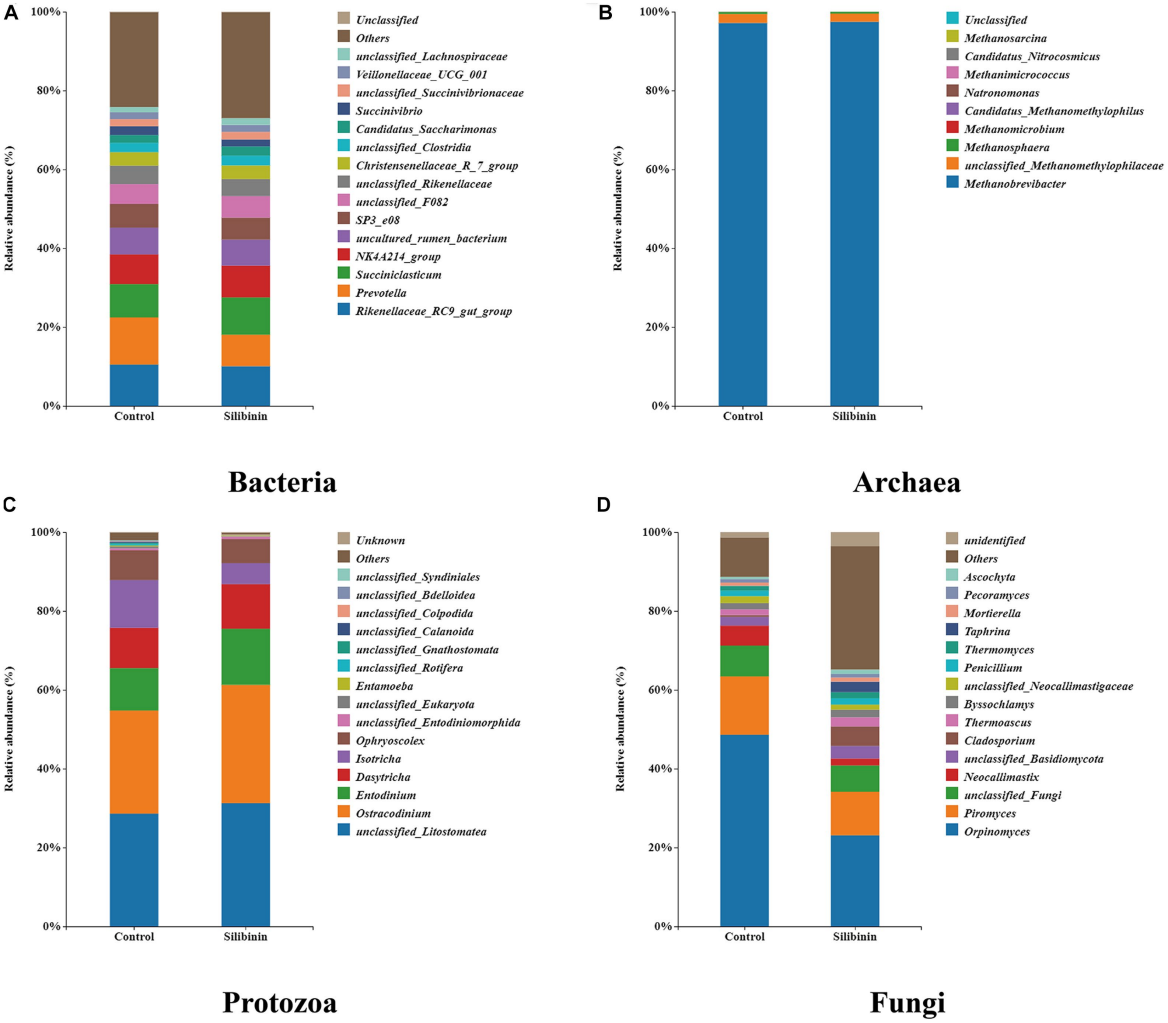
Relative abundance at the genus level of rumen microbiome in the 0 g/L and 0.60 g/L silibinin addition groups. **(A)** Bacteria relative abundance at the genus level. **(B)** Archaea relative abundance at the genus level. **(C)** Protozoa relative abundance at the genus level. **(D)** Fungi relative abundance at the genus level.

To further investigate changes in the rumen microbiome after the addition of silibinin, we select the control group and 0.60 g /L silibinin group, a total of 8 samples. 16S amplicon sequencing was performed by high-throughput sequencing for the rumen microbiome in the control and 0.60 g/L silibinin groups, and the α-diversity and β-diversity of the microbiome, as well as the relative abundance at the genus level, were analyzed. The microbial community diversity of bacteria, archaea, and protozoa in the 0.60 g/L silibinin group was not significantly different from the control group, but Simpson (*p* = 0.02) and Shannon (*p* = 0.02) of fungi were significantly different and higher than the untreated group (Table 4). There were also differences in the microbial community structure of bacteria, archaea, protozoa, and fungi in the rumen between the two groups (Figure 1). Figure 2 presents a visual representation of the relative abundance of species at the genus level for both the control group and the 0.60 g/L silibinin group. As the results of the relative abundance of rumen microbiome showed (Table 5), with the addition of 0.60 g/L silibinin, *Prevotella*, *Methanosphaera*, *Isotricha*, *Ophryoscolex*, and *unclassified_Rotifera*, *Orpinomyces*, *Neocallimastix* were significantly (*p* < 0.05) less in relative abundance than controls, while *Succiniclasticum*, *NK4A214_group*, *Candidatus_Saccharimonas*, *unclassified_Lachnospiraceae*, and *Others* were significantly more abundant than the control group (*p* < 0.05). These results indicated that supplementation with high doses of silibinin led to alterations in the ruminal microbial community, including changes in its structure and relative abundance.

**Table 4:** Effect of silibinin on the diversity of the most predominant microbial genera in the rumen after 24 h of *in vitro* incubation (%).

	CON^a^	SLB^b^	SEM	*p*-value
Bacteria				
ACE^c^	1202.48	1232.18	15.71	0.23
Chao1	1197.49	1227.25	15.55	0.22
Simpson	0.99	0.99	0.00030	0.48
Shannon	8.29	8.33	0.023	0.34
Archaea				
ACE	32.09	31.08	1.01	0.51
Chao1	31.75	30.75	0.99	0.50
Simpson	0.72	0.74	0.0093	0.14
Shannon	2.48	2.50	0.034	0.57
Protozoa				
ACE	115.32	90.24	0.00020	0.27
Chao1	114.63	89.01	14.46	0.26
Simpson	0.89	0.87	0.010	0.15
Shannon	4.14	3.85	0.14	0.18
Fungi				
ACE	135.63	286.89	60.46	0.13
Chao1	134.20	286.10	60.68	0.13
Simpson	0.89	0.95	0.015	0.022
Shannon	4.51	5.95	0.34	0.024

a Con = 0 g/L.

b SLB = 0.60 g/L.

c ACE = Abundance-based coverage estimator.

**Table 5:** Effect of silibinin on the relative abundance of rumen microorganisms after 24 h of *in vitro* fermentation (%).

Rumen microbiome	CON	SLB	*P*-value
Bacteria			
*Prevotella*	11.97 ± 1.43	7.96 ± 0.55	*
*Rikenellaceae_RC9_gut_group*	10.49 ± 0.67	10.09 ± 0.63	NS
*Succiniclasticum*	8.43 ± 0.62	9.46 ± 0.11	*
*NK4A214_group*	7.54 ± 0.13	8.07 ± 0.41	*
*uncultured_rumen_bacterium*	6.80 ± 0.48	6.62 ± 0.37	NS
*SP3_e08*	5.99 ± 0.77	5.58 ± 0.22	NS
*unclassified_F082*	5.05 ± 0.27	5.47 ± 0.26	NS
*unclassified_Rikenellaceae*	4.68 ± 0.42	4.31 ± 0.32	NS
*Christensenellaceae_R_7_group*	3.41 ± 0.15	3.46 ± 0.19	NS
*unclassified_Clostridia*	2.35 ± 0.14	2.45 ± 0.30	NS
*Succinivibrio*	2.24 ± 0.21	1.80 ± 0.25	NS
*Candidatus_Saccharimonas*	1.99 ± 0.068	2.33 ± 0.13	*
*unclassified_Succinivibrionaceae*	1.80 ± 0.35	1.91 ± 0.20	NS
*Veillonellaceae_UCG_001*	1.73 ± 0.077	1.78 ± 0.13	NS
*unclassified_Lachnospiraceae*	1.34 ± 0.10	1.71 ± 0.085	*
Protozoa			
*unclassified_Litostomatea*	28.57 ± 2.42	31.25 ± 1.93	NS
*Ostracodinium*	26.22 ± 3.98	30.02 ± 3.90	NS
*Isotricha*	12.16 ± 2.96	5.38 ± 1.23	*
*Entodinium*	10.72 ± 2.00	14.22 ± 1.47	NS
*Dasytricha*	10.24 ± 2.01	11.28 ± 4.01	NS
*Ophryoscolex*	7.52 ± 0.80	6.13 ± 0.93	*
*unclassified_Entodiniomorphida*	0.50 ± 0.0076	0.57 ± 0 0.13	NS
*unclassified_Eukaryota*	0.50 ± 0.68	0.15 ± 0.063	NS
*unclassified_Rotifera*	0.39 ± 0.76	0.00 ± 0.00	*
*unclassified_Gnathostomata*	0.27 ± 0.50	0.029 ± 0.070	NS
*unclassified_Calanoida*	0.25 ± 0.40	0.041 ± 0.047	NS
*Entamoeba*	0.23 ± 0.19	0.19 ± 0.10	NS
*unclassified_Bdelloidea*	0.18 ± 0.36	0.00 ± 0.00	NS
*unclassified_Colpodida*	0.12 ± 0.12	0.10 ± 0.089	NS
*unclassified_Syndiniales*	0.12 ± 0.16	0.045 ± 0.055	NS
Methanogens			
*Methanobrevibacter*	97.16 ± 0.46	97.46 ± 0.28	NS
*unclassified_Methanomethylophilaceae*	2.28 ± 0.50	2.09 ± 0.25	NS
*Methanosphaera*	0.51 ± 0.030	0.42 ± 0.051	*
*Methanomicrobium*	0.033 ± 0.027	0.010 ± 0.010	NS
*Candidatus_Methanomethylophilus*	0.0045 ± 0.0070	0.0093 ± 0.0069	NS
*Candidatus_Nitrocosmicus*	0.0025 ± 0.0051	0.0000 ± 0.0000	NS
*Natronomonas*	0.0021 ± 0.0032	0.0021 ± 0.0021	NS
*Methanimicrococcus*	0.0021 ± 0.0025	0.00084 ± 0.0017	NS
*Methanosarcina*	0.00082 ± 0.0016	0.0000 ± 0.0000	NS
Fungi			
*Orpinomyces*	48.61 ± 8.99	23.52 ± 16.44	*
*Piromyces*	14.8 ± 9.38	11.23 ± 8.56	NS
*unclassified_Fungi*	7.82 ± 2.68	6.72 ± 3.48	NS
*Neocallimastix*	5.06 ± 3.18	1.77 ± 1.11	*
*unclassified_Basidiomycota*	2.23 ± 1.22	3.25 ± 2.38	NS
*unclassified_Neocallimastigaceae*	1.76 ± 1.18	1.31 ± 0.92	NS
*Byssochlamys*	1.6 ± 0.68	1.96 ± 1.47	NS
*Thermoascus*	1.43 ± 0.87	2.35 ± 1.72	NS
*Penicillium*	1.37 ± 0.55	1.59 ± 0.49	NS
*Thermomyces*	1.24 ± 1.29	1.59 ± 1.72	NS
*Pecoramyces*	0.89 ± 0.67	0.88 ± 0.91	NS
*Mortierella*	0.84 ± 0.99	1.04 ± 0.73	NS
*Ascochyta*	0.55 ± 0.44	1.16 ± 1.39	NS
*Cladosporium*	0.48 ± 0.42	4.78 ± 7.13	NS
*Taphrina*	0.00069 ± 0.0008	2.54 ± 5.07	NS

^**^p < 0.01, ^*^p < 0.05, NS: notsignificant.

### 3.4. Effect of silibinin on rumen microbial metabolites and their KEGG pathway

**Figure 3:**
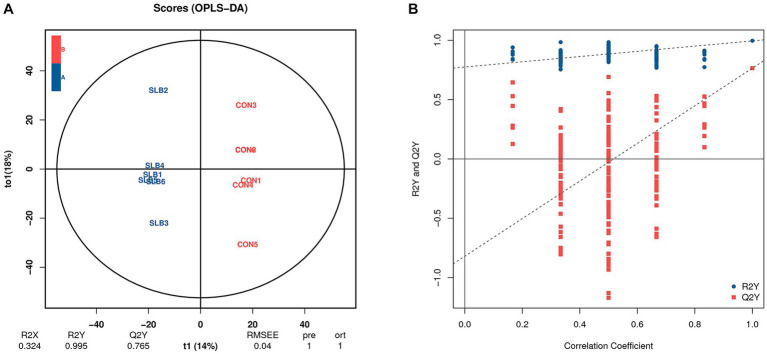
Orthogonal partial least squares-discriminant analysis (OPLS-DA) of the differences in rumen metabolites between 0 g/L and 0.60 g/L silibinin supplementation groups. **(A)** OPLS-DA score plot. **(B)** OPLS-DA model substitution test plot.

**Figure 4:**
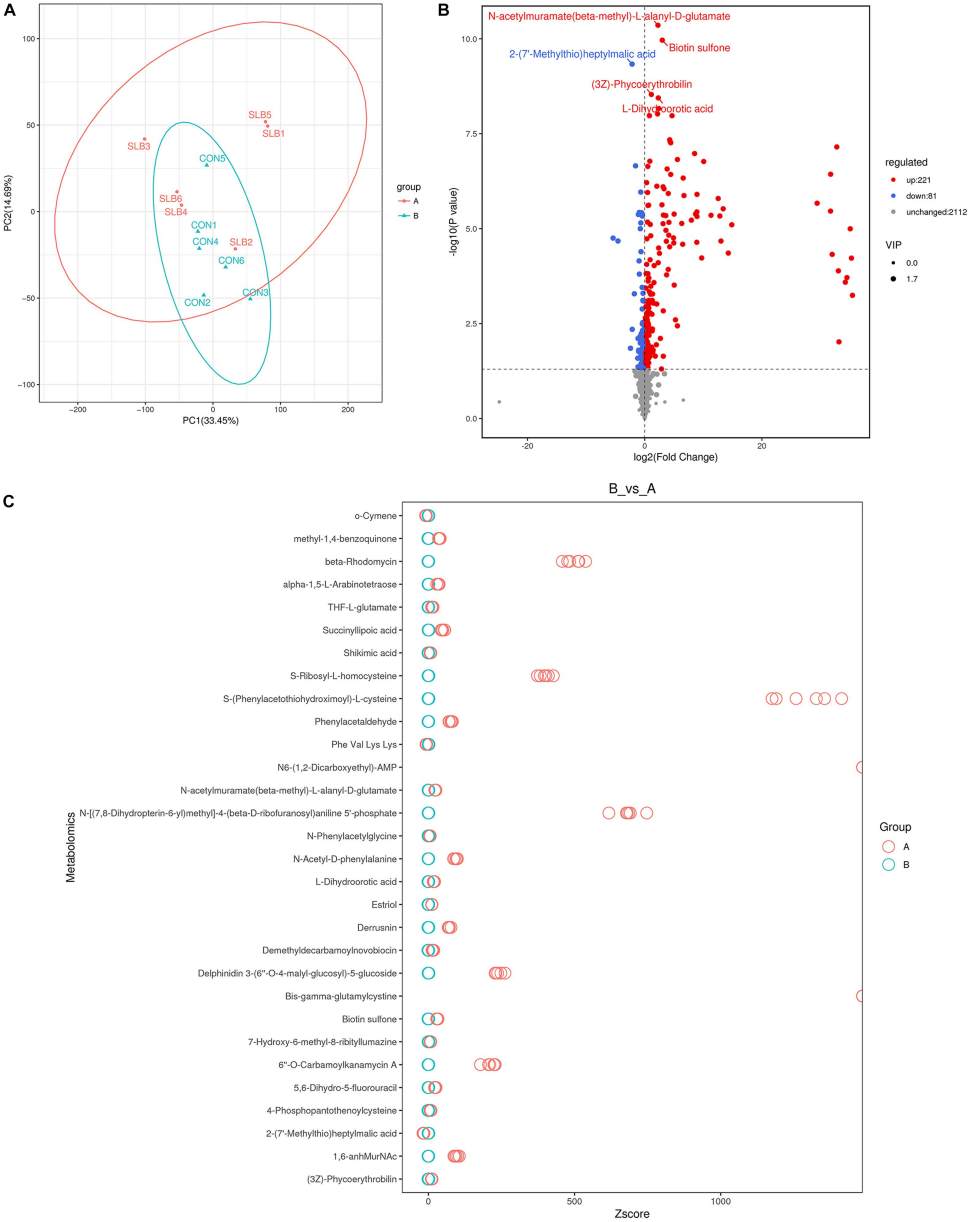
Screening of differential metabolites of rumen metabolites in 0 g/L and 0.60 g/L silibinin supplementation groups (A:SLB; B:CON). **(A)** Principal component analysis plots for differential groupings, When the number of repetitions in the group was greater than 3, the PCA plot showed the 95% elliptical confidence interval of the group. **(B)** Heat map of differential metabolite volcanoes, The blue points in the figure represent the down-regulated differentially expressed metabolites, the red points represent the upregulated differentially expressed metabolites, and the gray represents the metabolites detected but the difference is not significant. **(C)** Differential metabolism Z-score plot, each hollow circle represents the Z-score of the metabolite content in a sample, and the hollow circle color represents different groups.

Rumen metabolites were analyzed by non-targeted metabolic means to investigate the association between the rumen microbiome and rumen-associated metabolites. A total of 9,162 peaks were detected in 12 samples for qualitative and quantitative metabolism analysis, with 2,414 metabolites annotated for metabolites detected in default mode. The model was evaluated using orthogonal projections to latent structure-discrimination analysis (OPLS-DA) with R^2^Y = 0.995 and Q^2^Y = 0.789, which is close to 1, indicating that the model is more stable and reliable, i.e., it can be used to screen for differential metabolites. To check the reliability of the OPLS-DA model, we performed a permutation test. A positive slope of the Q^2^Y fitted regression line indicates that the model is meaningful. The blue dots are generally above the red dots, indicating that the model’s training and test sets are independent (Figure 3). The OPLS-DA model can be used to identify differences between the two metabolite groups. Based on the results of OPLS-DA, the differential metabolites screened from the variable importance in projection (VIP) of the obtained multivariate analysis OPLS-DA model, combined with fold change and *p*-value (VIP > 1, *p* ≤ 0.05, FC > 1 or FC < 0.05) were considered as rumen differential metabolites between the two groups (Table 6). Through conducting a principal component analysis on the samples, we can gather initial insights into the systemic metabolic differences between the control and 0.60 g/L silibinin groups (Figure 4A). The volcano plot (Figure 4B) depicts the degree of difference in metabolite levels between the two groups. Each point on the plot indicates a different metabolite, and its position is determined by the *p*-value (log base 10) represented on the y-axis, as well as fold change (log base 2) shown on the x-axis. The size of each point is determined by its VIP (Variable Importance in Projection) value for an OPLS-DA model, indicating the significance of differential expression screening with larger scattered points reflecting more reliable differentially expressed metabolites. The plot shows 221 upregulated differentially expressed metabolites represented by red dots and 81 down-regulated differentially expressed metabolites represented by blue dots. Gray dots represent metabolites detected but not significantly different, indicating no significant change of 2,112 metabolites. After sorting by *p*-value, the top 5 metabolites with significant differences compared to the control group were labeled in the graph. Among them, N-acetylmuramate(beta-methyl)-L-alanyl-D-glutamate, Biotin sulfone, (3Z)-Phycoerythrobilin, L-Dihyd oorotic acid were significantly upregulated (*p* < 0.05) whereas 2-(7′-methylthio)heptylmalic acid was down-regulated (*p* < 0.05). Similarly, the Z-score (standard score) based on the quantitative values of metabolites is used to measure the deviation of differences between the experimental group and the control group. The Z-score plot displays the top 30 differential metabolites sorted by *p*-value, providing an intuitive comparison of metabolite differences between the two groups. Each hollow circle represents the Z-score of metabolite levels in each sample, with the color of the hollow circles representing different groups (Figure 4C).

**Table 6:** Significant differences in metabolic between the control and 0.60 g/L silibinin groups.

ID^a^	Description	FC^b^	*p*-value	VIP^c^	Up or down
pos_2562	2-Phenylacetamide	1.215019467	0.003176788	2.124390797	up
neg_2885	Phlorizin	2553.268079	0.000004441	2.604237268	up
pos_2536	Dalspinin	5954.317677	0.000001606	2.61583266	up
pos_2548	N6-(1,2-Dicarboxyethyl)-AMP	7,357,251,514	0.000000070	2.638870091	up
neg_3440	5,6,7,8-Tetrahydromethanopterin	9,539,211,917	0.009557470	2.050420297	up
pos_2520	FMNH^d^	3,516,880,248	0.000003450	2.628961025	up
pos_2549	Pyridoxine 5′-phosphate	721657510.3	0.000002136	2.635698761	up
pos_2517	Silibinin	8290.016956	0.000021214	2.569872104	up
pos_2557	Beta-D-Fructose 6-phosphate	29745.70802	0.000007900	2.625104301	up

a ID = Identity document.

b FC = Fold change.

c VIP = Variable Importance in Projection.

d FMNH = Flavin mononucleotide adenine dinucleotide reduced form.

Note: The first column is the ID of the metabolite, the second column is the English name of the metabolite, and in the following order: fold of difference, *p* value for univariate analysis, VIP value for OPLS-DA model, and up- and down-regulation information.

As different metabolites interact in the organism, different pathways are formed. To gain a more comprehensive understanding of the impact of silibinin on rumen metabolic pathways, we identified and screened for metabolic pathways that displayed significant differences between the two groups. Table 7 showed that the 0.60 g/L silibinin group differed significantly from the control group in the phenylalanine metabolic pathway (*p* = 0.02), and there was a trend of difference in two metabolic pathways: flavonoid biosynthesis (*p* = 0.08) and folate biosynthesis (*p* = 0.10).

**Table 7:** Significant differences in metabolic pathways between the control and 0.60 g/L silibinin groups.

ID^a^	Description	Metabolite Ratio	Bg Ratio	Enrich factor	*p*-value	Q-value
ko00360	Phenylalanine metabolism	6.25%	1.82%	3.44	0.019431	0.784349
ko00941	Flavonoid biosynthesis	3.12%	0.81%	3.87	0.083115	0.784349
ko00790	Folate biosynthesis	4.69%	1.82%	2.58	0.097809	0.784349

a ID = Identity document.

We screened for differential metabolites in the control and 0.60 g/L silibinin groups with FC < 0.05 or FC > 1 and *p* ≤ 0.01 (Table 6). 2-Phenylacetamide, N6-(1,2-Dicarboxyethyl)-AMP, phlorizin, Dalspinin, 5,6.7.8-Tetrahydromethanopterin, FMNH, Pyridoxine 5′-phosphate, Silibinin, Beta-D-Fructose 6-phosphate were all up-regulate.

## 4. Discussion

In this experiment, based on the linear effects of silibinin on most indicators, we selected the maximum dosage group as the research subject for analyzing 16S amplicons and non-targeted metabolomics. By comparing this group with the control group, we aimed to investigate the impact of silibinin on the rumen microbial community, metabolic products, and metabolic pathways to unveil its mechanism in regulating rumen gas production and fermentation parameters. After thoroughly analyzing the results, we discovered that high-dose silibinin influences methane production in the rumen directly and indirectly.

First, an indirect effect of silibinin is its ability to reduce the concentration of methane precursors, thereby indirectly influencing methane production. Ruminal methane production results from the combined effect of various microorganisms within the rumen, and microorganisms, such as protozoa, bacteria, and fungi, play important roles in methane generation within the rumen (Hook et al., 2010). In the rumen, almost all ciliated protozoa are associated with methane-producing bacteria; there is a portion of methane-producing bacteria in the cytoplasm of rumen ciliate protozoa, forming a mutualistic or symbiotic relationship between them (Finlay et al., 1994) which facilitates methanogens in utilizing the H_2_ produced by the protozoa to generate methane. *Isotricha* and *Ophryoscolex* belong to ciliate protozoa (Schrenk and Bardele, 1987; Ivan et al., 2000), and in this experiment, compared to the control group, the relative abundance of these two protozoa was significantly reduced, which may be one of the reasons for the significant decrease in *Methanosphaera*. Related research has also shown that plant extracts rich in flavonoids have an inhibitory effect on rumen ciliate protozoa (Kim et al., 2015), consistent with the results of this experiment. Furthermore, rumen ciliate protozoa can adhere to feed particles and degrade cellulose, hemicellulose, and starch (Williams et al., 1984; Mendoza et al., 1993), ultimately producing CO_2_ and H_2_ for methanogens to utilize. Additionally, the absence of protozoa has been found to reduce organic matter degradation (Newbold et al., 2015). Moreover, silibinin, chemically belonging to flavonoids, has been found to disrupt the cell membrane of rumen protozoa and reduce their population (Ku-Vera et al., 2020), thereby reducing hydrogen transfer between protozoa and methane-producing bacteria. Based on these findings, we speculate that high doses of silibinin may have acted in a similar way on protozoa, leading to the decreased relative abundance of *Isotricha* and *Ophryoscolex* observed. This decrease in abundance could have resulted in changes in metabolite production and a reduction in the concentrations of CO_2_ and H_2_, ultimately affecting methane levels (Ranilla et al., 2007).

In addition, other indirect effects of silibinin include increased synthesis of propionate and reduced methane production by competitively utilizing substrate H_2_. Bacteria and fungi in the rumen play a crucial role in fiber degradation, leading to an increase in dry matter degradation rate and the production of VFAs, CO_2_, and H_2_. After adding high doses of silibinin, we observed a significant increase in the relative abundance of *Succiniclasticum* and *NK4A214_group*. *Succiniclasticum* has been noted to promote the metabolism of succinate derived from carbohydrate fermentation into propionate (Van Gylswyk, 1995). The production of propionate reduces H_2_ partial pressure and ensures normal rumen fermentation. However, the relationship between *NK4A214_group* and propionic acid production remains unclear, and its specific role in the rumen is still not well understood (Pacifico et al., 2021). Further, N6-(1,2-Dicarboxyethyl)-AMP, an upregulated differential metabolite, can be converted into fumarate, which is a precursor of propionate metabolism, by adenosylsuccinate lyase (Timpani et al., 2020). Fumarate acts as an alternative hydrogen sink in the rumen and competes with methanogenic bacteria for H_2_, thereby reducing CH_4_ production (Newbold and Rode, 2006). Based on the above analysis, we speculate that the increase in the proportion of propionate in the rumen is likely attributable to the increased relative abundance of *Succiniclasticum*. It is equally important to note that after adding a high dose of silibinin, there was a significant decrease in the relative abundances of *Prevotella*, *Orpinomyces*, and *Neocallimastix*. Previous studies have shown that flavonoids are usually present in their glycoside form, which is further deglycosylated by glycosidases secreted by microbial communities, increasing aglycone concentrations (Murota et al., 2018); bacteria have evolved diverse metabolic lifestyles and strategies to harvest energy from glycosides (Wang et al., 2022). Moreover, compared to the Proteobacteria phylum, microorganisms within the Bacteroidetes phylum are selectively inhibited by flavonoids (Yu et al., 2023). This explains the increased relative abundances of *Succiniclasticum* and *NK4A214_group* and the decreased relative abundance of *Prevotella* after adding a high dose of silibinin. Furthermore, *Orpinomyces* and *Neocallimastix* ferment CO_2_ and H_2_ as end products (Williams et al., 2020), and their relative abundances significantly decrease, reducing not only the proportions of CO_2_ and H_2_ but also indirectly lowering methane concentrations. In summary, we speculate that high doses of silibinin regulate the relative abundances and metabolic products of bacteria and fungi in the rumen, affecting the concentration of methane precursors and promoting propionate synthesis through competitive utilization of H_2_, ultimately leading to a reduction in methane production (Zhou et al., 2021).

The direct effect of silibinin in reducing methane production involves modulating the activity of rumen methanogenic bacteria. Ruminal methanogenic bacteria utilize the reducing power generated through rumen fermentation to reduce carbon dioxide, formic acid, methanol or methylamine to CH_4_. This study showed that compared to the control group, the relative abundance of *Methanosphaera* was significantly reduced after adding a high dose of silibinin. It is worth noting that *Methanosphaera* accounts for a relatively small proportion and may have a limited effect on reducing methane concentration. Previous reports have also found that flavonoids can inhibit the activity of *Methanosphaera* and other methanogenic bacteria and thus reduce CH_4_ emissions (Seradj et al., 2014; Hassan et al., 2020). Furthermore, epicatechin, quercetin, isoquercetin, and luteolin-7-glucoside reduced the ratio of CH_4_ production to total gas production (Sinz et al., 2018). In this study, the percentage of methane in the total gas production also significantly decreased with increasing doses of silibinin, consistent with previous research findings.

Furthermore, after analyzing the metabolites, we found that flavin mononucleotide adenine dinucleotide reduced form (FMNH), Pyridoxine 5′-phosphate, and 5,6,7,8-Tetrahydromethanopterin exhibited upregulation. FMNH plays a role in the electron transfer during methane production (Thauer et al., 2008), ultimately being reduced to FMNH_2_. Pyridoxine 5′-phosphate can be converted into pyridoxal 5′-phosphate (Garrido-Franco, 2003), which inhibits the activity of the final enzyme involved in methane synthesis, namely coenzyme M methyltransferase (Van Der Meijden et al., 1983). During methane generation, 5,6,7,8-Tetrahydromethanopterin (H_4_MPT) acts as a carrier for the formyl group through the methyl-reduced C1 unit (Van Beelen et al., 1984), forming 10-formyl-H_4_MPT in methylotrophic organisms (Keltjens et al., 1990). We speculate that it may be that *Methanosphaera*, a methylotrophic pathway archaea (Wolin, 1985), whose relative abundance significantly decreased, lead to the obstruction of CH_4_ synthesis and resulting in the upregulation of intermediates such as FMNH, Pyridoxine 5′-phosphate, and 5,6,7,8-Tetrahydromethanopterin. However, further research is needed to validate this. In addition to impacting the production of metabolites, the addition of 0.60 g/L silibinin also had an effect on the phenylalanine metabolic pathway. We presume that the high dose of silibinin affecting the phenylalanine metabolism resulted in the upregulation of differential metabolites 2-Phenylacetamide, Phlorizin, and Dalspinin. In summary, we hypothesize that silibinin modulates the relevant pathways and metabolites in the rumen in a way that affects methane production in the rumen and reduces the proportion of CH_4_.

In addition, this experiment found that the concentration of NH_3_-N did not show a linear decrease with increasing dosage of silibinin, and only the group with 0.60 g/L silibinin showed a significant reduction in NH_3_-N concentration. Previous reports have also shown that supplementation of citrus flavonoids, flavones, myricetin, naringin, catechin, rutin, quercetin, and kaempferol does not affect rumen NH_3_-N concentration (Oskoueian et al., 2013; Amin et al., 2021). Additionally, *Prevotella* is involved in the degradation of plant-derived semi-fibrous materials and participates in the process of protein degradation (Betancur-Murillo et al., 2022). We speculate that the decrease in *Prevotella* relative abundance, possibly due to the addition of 0.60 g/L silibinin, leads to a reduction in protein degradation and subsequently decreases NH_3_-N concentration (Zhan et al., 2017). However, the optimal range for rumen microbial growth is between 10–50 mg/dL of NH_3_-N concentration (Firkins et al., 2007). In this experiment, the rumen NH_3_-N concentration was within this range, indicating that adding a high dose of silibinin may not have a negative impact on microbial biomass protein synthesis. Moreover, related studies have shown a negative correlation between the relative abundance of rumen family *NK4A214_group* and NH_3_-N concentration (Liu et al., 2022), as well as a positive correlation between protozoa and NH_3_-N concentration (Wanapat and Pimpa, 1999), which is consistent with the results of this study. These findings provide new insights into the role of silibinin in regulating the rumen microbial community and methane production mechanisms. On the other hand, from the experimental results, the ingestion of a high dose of silibinin caused a decrease in dry matter degradation rate, which is likely the main reason for the decrease in total volatile fatty acid concentration, and, consequently, the pH value would correspondingly increase. Relevant literature has also indicated that supplementing mulberry leaf flavonoids did not affect the apparent digestibility of dry matter and organic matter (Chen et al., 2015). These results suggest that different plant-derived flavonoid compounds may produce different effects at varying concentrations. In ruminant animal production, volatile fatty acids are important sources of energy, and changes in VFA concentration can affect milk production in cows (Morvay et al., 2011). Therefore, the aim of our study is to reduce methane production while ensuring cow productivity. In this experiment, we selected the group supplemented with 0.60 g/L of silibinin for analysis to better observe the effect of silibinin on rumen methane production. Compared to the group supplemented with 0.00 g/L of silibinin, the group supplemented with 0.075 g/L of silibinin showed no significant difference in total VFA concentration but a significant decrease in methane concentration. Based on this, it can be preliminarily concluded that adding 0.075 g/L of silibinin *in vitro* experiments can reduce methane concentration without affecting rumen fermentation. Therefore, this group can serve as an additive for *in vivo* experiments to evaluate the methane-inhibiting effect of silibinin in living organisms.

## 5. Conclusion

This study provides a theoretical basis for the application of a novel rumen methane regulator. In an *in vitro* fermentation experiment, silibinin modulates rumen microbial community diversity and community structure, leading to changes in rumen metabolic pathways and metabolites to reduce methane emissions. Notably, silibinin demonstrated the ability to lower methane emissions at reasonable doses without adversely impacting rumen fermentation. Nevertheless, further verification through long-term *in vivo* experiments is crucial in light of the possible adaptation of rumen microbes to metabolites and differences between *in vitro* and *in vivo* findings.

## Data availability statement

The data presented in the study are deposited in the NCBI Sequence Read Archive (SRA) repository, accession number PRJNA975466 and PRJNA976941. https://www.ncbi.nlm.nih.gov/sra.

## Ethics statement

The animal use protocol was approved following the Animal Care and Use Committee of Northeast Agricultural University (protocol number: NEAUEC20230268).

## Author contributions

RL and YS: methodology, investigation, and writing—original draft. HM, ML, BD, WS, and YQ: investigation and data curation. YL and BD: conceptualization, methodology, resources, funding acquisition, and writing—review and editing. YZ: writing—review and Editing. All authors contributed to the article and approved the submitted version.

## Funding

This study was financially supported by the Natural Science Foundation of Heilongjiang Province (YQ2023C011), Key Research and Development Program of Heilongjiang Province of China (grant no. 2022ZX01A24), “Academic Backbone” Project of Northeast Agricultural University (22XG35) and Key Laboratory of Low-carbon Green Agriculture in Northeastern China, Ministry of Agriculture and Rural Affairs P. R. China (grant no. LCGANE14).

## Conflict of interest

YS was employed by Beijing Sunlon Livestock Development Company Limited.

The remaining authors declare that the research was conducted in the absence of any commercial or financial relationships that could be construed as a potential conflict of interest.

## Publisher’s note

All claims expressed in this article are solely those of the authors and do not necessarily represent those of their affiliated organizations, or those of the publisher, the editors and the reviewers. Any product that may be evaluated in this article, or claim that may be made by its manufacturer, is not guaranteed or endorsed by the publisher.
